# COVID-19

**DOI:** 10.47626/1679-4435-2021-201

**Published:** 2022-03-30

**Authors:** Rosylane Rocha

**Affiliations:** President of the National Association of Occupational Medicine

The first known infection of COVID-19, caused by severe acute respiratory syndrome coronavirus 2 (SARS-CoV-2), occurred on November 17, 2019, according to data from the Chinese government. The patient was a 55-year-old person from Hubei province, near Wuhan, the focus of the first outbreak.^1^ On February 26, 2020, the first case of COVID-19 was identified in Brazil.^2^

On January 30, 2020,^1^ the World Health Organization (WHO) declared the outbreak of the novel coronavirus a public health emergency of international concern - the highest alert level of the organization, as foreseen in the International Health Regulations.

Since then, several researchers have developed studies with the purpose of better understanding the novel coronavirus and the clinical and epidemiological aspects of the disease. It turned out to be an insane though heroic quest in an unequal confrontation with an “invisible” enemy that, in a cruel and fast way, was taking the lives and joy of millions of people and their families around the world.

It was not until March 11, 2020, that the WHO characterized COVID-19 as a pandemic. Since then, hundreds of journals, with different impact factors, have received a plethora of scientific articles on COVID-19. The publications have several scientific qualities and research methodologies with a variety of levels of evidence.

In the race against time and with the need to publish original studies that would disclose the adequate clinical management and cure for such a terrible disease, necessary steps for research analysis were skipped in some cases, which ended up revealing a certain fragility in the submissions.

The scientific community was surprised by the publication and dissemination of articles reporting findings that caught the world’s attention, but which, after peer review, were retracted and much criticized. Thus, it is very important not to skip steps in order to expedite the manuscript submission and publication process.

In such dark times and even with many difficulties in the field of scientific research, Brazil has stood out. A study published in the Annals of the Brazilian Academy of Sciences analyzed 61,000 articles between January 1 and December 13, 2020, indexed in the Web of Science database.^3^ In 2020, 4 countries - the United States (28%), China, Italy, and England (10% to 12% each) - accounted for 60% of all scientific output on COVID-19. In Latin America, Brazil was the country with the highest scientific output and publications: 2,582 articles were published (about 4%), being part of the group of 12 countries that accounted for 95% of the world’s scientific output.

Despite the advance of vaccination and its indisputable effect on stopping deaths, COVID-19 still poses great challenges to be overcome. Many studies still need to be conducted and published, not only for the moment we are living through but also for the future as a historical and scientific record of the biological warfare that took place and led to the death of approximately 6 million people worldwide.

Our Brazilian Journal of Occupational Medicine (*Revista Brasileira de Medicina do Trabalho*) presents studies of scientific relevance in this special issue.

Enjoy your reading!
